# Effects of auditory distraction on voluntary movements: exploring the underlying mechanisms associated with parallel processing

**DOI:** 10.1007/s00426-017-0859-5

**Published:** 2017-04-08

**Authors:** Marcelo Bigliassi, Costas I. Karageorghis, Alexander V. Nowicky, Michael J. Wright, Guido Orgs

**Affiliations:** 1 Department of Life Sciences, Brunel University London, Kingston Lane, Uxbridge, Middlesex UB8 3PH UK; 2 Department of Clinical Sciences, Brunel University London, Kingston Lane, Uxbridge, Middlesex UB8 3PH UK; 3 Department of Psychology, Goldsmiths, University of London, 8 Lewisham Way, London, SE14 6NW UK

## Abstract

Highly demanding cognitive-motor tasks can be negatively influenced by the presence of auditory stimuli. The human brain attempts to partially suppress the processing of potential distractors in order that motor tasks can be completed successfully. The present study sought to further understand the attentional neural systems that activate in response to potential distractors during the execution of movements. Nineteen participants (9 women and 10 men) were administered isometric ankle-dorsiflexion tasks for 10 s at a light intensity. Electroencephalography was used to assess the electrical activity in the brain, and a music excerpt was used to distract participants. Three conditions were administered: auditory distraction during the execution of movement (auditory distraction; AD), movement execution in the absence of auditory distraction (control; CO), and auditory distraction in the absence of movement (stimulus-only; SO). AD was compared with SO to identify the mechanisms underlying the attentional processing associated with attentional shifts from internal association (task-related) to external (task-unrelated) sensory cues. The results of the present study indicated that the EMG amplitude was not compromised when the auditory stimulus was administered. Accordingly, EEG activity was upregulated at 0.368 s in AD when compared to SO. Source reconstruction analysis indicated that right and central parietal regions of the cortex activated at 0.368 s in order to reduce the processing of task-irrelevant stimuli during the execution of movements. The brain mechanisms that underlie the control of potential distractors during exercise were possibly associated with the activity of the frontoparietal network.

## Introduction

Selective attention is among the most fundamental and important functions of the human brain (Driver 2001). In 1958, Daniel Broadbent proposed that attention allocation was generally defined by the physical features of an environmental signal. Broadbent’s filter model had enormous impact on the scientific world, given that its main postulate was that only relevant signals are processed by the brain. Broadbent’s assertion was predicated on the fact that the brain has limited capacity to process sensory signals from multiple sources; thus, strong sensory signals from one’s environment were hypothesized to force attention towards external influences. For example, the sound constituents of loudness and pitch can elicit rapid shifts of attention (see Lee et al. 2013), given that they are directly relevant to the survival of the organism (Walker, Bizley, King, & Schnupp 2011).

The human cortex is able to process the physical features of a range of stimuli and initiate actions that are predicated on the stimulus relevance. The sound of an explosion nearby immediately reallocates one’s attentional focus toward auditory pathways in order to initiate an action that mitigates any potential harm (Petersen & Posner 2012). In such instances, auditory stimuli appear to initiate cascade reactions that activate brain regions (e.g. amygdala and hypothalamus) that are associated with survival functions (see LeDoux 2012). Irrelevant stimuli, on the other hand, are routinely dismissed through a process of sensory blockage (Mysore & Knudsen 2013). The blocking of such stimuli allows humans to focus more intently on task-relevant information, avoiding the influence of potential distractors that might compromise task performance (e.g. Garrison & Williams 2013).

During the execution of movements, the brain attempts to select the most salient signals and duly allocate the most attentional capacity toward them in order to complete a given task successfully (Hutchinson & Tenenbaum 2007). However, there are multifarious internal and external sensory cues during conditions of physical effort (Katsuki & Constantinidis 2014). The muscles, heart, and lungs emit signals to the brain as a means by which to facilitate one’s sense of exertion (Noakes, Clair Gibson, & Lambert 2005). In order to cope with the prophylactic influences of interoceptive sensory cues, the brain needs to engage in dissociative strategies (e.g. directing attention to surrounding scenery). The reallocation of attentional focus towards task-unrelated information (e.g. an aesthetically pleasing landscape) allows fatigue-related symptoms (e.g. limb discomfort) to remain outside of focal awareness and renders the execution of simple movements partially automatic (Kal, Kamp, & Houdijk 2013; Lohse & Sherwood 2012; McNevin, Shea, & Wulf 2003). This is owing to the fact that interoceptive sensory cues are not sufficiently potent to increase the use of associative thoughts (Hutchinson, Karageorghis, & Jones 2015). However, the execution of complex movements usually requires high levels of concentration and generally entails only mild symptoms of fatigue, meaning that attentional focus has to be entirely allocated to task-related information (e.g. target-shooting performance; Pashler, Johnston, & Ruthruff 2001). In such instances, irrelevant stimuli need to be suppressed (Geng 2014) or processed in such a way that task performance is not compromised (i.e. parallel processing; see Rejeski 1985; Wilson, Vine, & Wood 2009).

Music-related interventions have been used in the field of exercise and sport as a means by which to promote the use of dissociative thoughts and improve exercise performance (see Karageorghis & Priest 2012a, b for review). However, the use of music is hypothesized to have a detrimental effect on motor performance if the exercise mode demands high levels of concentration. In such instances, the human brain attempts to partially suppress or parallel process potential distractors to enable the organism to engage with the task. The underlying mechanisms of parallel processing have been researched extensively in the field of visual sciences (for review, see Thornton & Gilden 2007), but remain uncharted during the execution of motor tasks (e.g. Bullock & Giesbrecht 2014).

## Processing of potential distractors

The human brain houses an extensive neural network that enables multifaceted connectivity between different areas, which leads to the manifestation of complex emotions and decisions (Bassett & Gazzaniga 2011). The brain is capable of processing and suppressing a variety of signals [i.e. internal (e.g. muscle afferents) and external (e.g. music) sensory cues] in tandem; this organ does not function on a *stop-and-go* basis. In actuality, there is a constant flow of information between peripheral and central nervous systems (Hernández-Peón, Brust-Carmona, Peñaloza-Rojas, & Bach-Y-Rita 1961). The parietal lobe of the brain has been identified to be the region responsible for selecting the most salient signals and informing other areas about the relevance of the signal (see Yantis 2008). However, other regions of the cortex such as the frontal lobe can also play a central role in the partial suppression of attentional distractors (Suzuki & Gottlieb 2013). The frontal and parietal lobes are integrated (Brunetti et al. 2008) and appear to operate in tandem as a means by which to define which pieces of information are most relevant (Ptak 2012).

The brain mechanisms associated with attentional shifts can be rendered more complex under exercise conditions when compared to a resting state; this is due to the fact that a larger proportion of the brain is active (Secher, Seifert, & Van Lieshout 2008). In order to generate movement, the primary motor cortex has to send neural messages to the spinal cord, which subsequently causes skeletal muscles to contract. Concurrently, the brain needs to process interoceptive (e.g. muscle afferents; Pollak et al. 2014) and environmental (e.g. visual information; Hutchinson et al. 2015) sensory cues. Thus, attentional mechanisms bear substantial influence over the gamut of factors that underlie task performance (Lohse & Sherwood 2012).

## Rationale for the present study

In 1985, Rejeski developed a theory predicated on Broadbent’s (1958) idea (i.e. attention depends on the physical features of the signal) to explain the integrative processes that take place when individuals need to cope with internal (bodily) and external (environmental) cues during movement execution. Rejeski advanced the parallel processing theory, which posits that interoceptive signals compete for attention with external, environmental cues because the brain is limited in its capacity to process sensory information from multiple sources. Accordingly, even strong sensory signals, as previously proposed by Broadbent (1958), could be partially suppressed given that internal cues are deemed to be more relevant than external influences during the execution of movements. This theory has been tested extensively in the field of exercise science by use of music as an ecologically valid stimulus that directs attention toward an external influence and ameliorates the effects of fatigue (see Karageorghis & Priest 2012a, b).

Subsequently, Tenenbaum (2001) suggested that attentional focus is moderated primarily by exercise intensity and complexity (e.g. Stroop test). His theoretical propositions advanced Broadbent’s idea and introduced a number of moderators (e.g. exercise intensity and mode) through which attention could be influenced during the execution of movements. During exercise, individuals are able to focus outwardly if task-related factors (e.g. muscle acidosis) do not enter focal awareness. Highly demanding motor tasks, in terms of intensity and/or complexity, have the potential to partially suppress task-irrelevant signals, such as environmental distractions. This is essentially a means by which attention can be guided inwardly.

Collectively, the theoretical contributions of Rejeski (1985) and Tenenbaum (2001) support the notion that sensory signals are processed by the brain in parallel channels, and that exercise intensity and complexity can moderate the degree to which attention is reallocated toward internal and external sensory cues. In the present context, the interaction of exercise intensity and complexity constitutes the “relevant signals” to which Broadbent (1958) was alluding. Accordingly, auditory distractions can disrupt task performance if the exercise mode and/or intensity demand high levels of concentration (Lavie 2005). In such instances, the brain appears to parallel process potential distractors in order to enable the organism to fully engage with the task at hand (Geng 2014). Put another way, attentional processes mediate the perception–action cycle as a means by which to facilitate movement execution (cf. Cutsuridis 2013). These attentional processes also have a direct effect on decision-making functions, allowing the organism to execute a motor plan and sustain neural control of working muscles (Lohse et al. 2011; Lohse & Sherwood 2012).

Once an external stimulus enters focal awareness, it could potentially disrupt an action and thus force the organism to rearrange the motor plan (e.g. Wood & Wilson 2010). Parallel processing and attentional suppression mechanisms can prevent the detrimental effects of sensory distraction on voluntary control when the physical features of the stimulus are not overbearing (Quartana et al. 2007). Strong sensory signals (e.g. startling auditory stimuli), on the other hand, can override this attentional threshold, enter focal awareness, and force individuals to reorganize the task, in accord with the new situational demands imposed by the stimuli (Hommel 2010).

## Aim of the present study

This was an exploratory study intended to further understand the neural systems that activate in response to auditory stimuli during the execution of attention-demanding tasks. It was hypothesized that highly demanding cognitive-motor tasks would guide attentional focus toward task-related information and reduce the processing of external influences to some extent. Parallel processing mechanisms were also hypothesized to prevent the disruption of the motor action and protect higher-order cognitive functions (i.e. reduced activity in the frontal lobe) against potential distractors (Suzuki & Gottlieb 2013). An auditory stimulus was used to manipulate attention and thus assist in furthering understanding of the attentional processes that underlie movement execution. An event-related potential (ERP) study was developed using a simple motor task (isometric ankle-dorsiflexion). Brain reconstruction analyses were used to identify the neural networks that activate to prevent sensory signals from entering focal awareness.

## Event-related potential analysis

Event-related potential experiments have been a common means to further understand attentional mechanisms associated with sensory stimulation (e.g. Popovich & Staines 2015). Attentional responses occur for very short periods of time and researchers generally require high-temporal resolution techniques to acquire meaningful data (Light et al. 2010). Reallocation of attentional focus from one source of sensory information to another has been repeatedly linked to changes in the electroencephalographic waveform (e.g. Rapela, Gramann, Westerfield, Townsend, & Makeig 2012; Spielmann, Schroger, Kotz, & Bendixen 2014). Luck, Woodman, & Vogel (2000) suggested that waveform changes caused by attention modulation might be moderated by situational demands and type of sensory information. For example, auditory distractions have been commonly associated with up/down modulations in the EEG waveform following ~300 s of stimulus onset. It has been hypothesized that P3 (~350 ms) is primarily induced by stimulus-driven attentional processes that originate in the frontal cortex (Polich 2007). Conversely, recent evidence indicates that up modulations in P3 could also be associated with stimulus evaluation and decision-making processes (e.g. Twomey, Murphy, Murphy, & O’Connell 2015). In the context of the present study, the reception of sensory stimuli during the execution of motor tasks might impose greater challenges for the brain and ERP components might differ owing to the amount of internal and external sensory information that needs to be processed in tandem.

## Research hypotheses

### Muscle electrical activity

It was hypothesized that the auditory stimulus would not modulate the neural activation of the working muscle and voluntary control because the brain could easily process potential distractors during a simple cognitive-motor dual task (Caputo & Guerra 1998; Geng 2014). Participants were expected to partially inhibit the processing of extremely irrelevant information as a means by which to execute the task successfully. Simple auditory distractions are hypothesized to be not sufficiently challenging to compromise the force produced and possibly ineffective in modulating the electrical activity in the muscles. In such instances, a parallel processing mechanism could be identified using the premise that participants are able to sustain the muscle contraction at the required level regardless of the presence of auditory distraction.

### Brain activity

When participants receive auditory stimulation at rest, processing of potential distractors has been shown to occur at approximately 300 ms after the stimulus onset (Horváth, Sussman, Winkler, & Schröger 2011; Polich 2007). However, the reception of sensory stimuli during the execution of motor tasks might impose greater challenges for the brain. In such instances, the evoked response could be influenced by task-related factors. We hypothesized that the presence of task-related factors, such as interoceptive sensory cues and visual feedback, would upregulate the electroencephalographic waveform at ~300 ms following stimulus onset to a greater degree than a no-exercise condition (cf. Berti & Schröger 2003). In order to cope with the detrimental effects of auditory distractions, participants would need to parallel process the stimulus in such a way that it does not enter focal awareness and disrupt task performance. Furthermore, the parietal and frontal lobes have been implicated in the processing of attentional distractors (Suzuki & Gottlieb 2013). The parietal and posterior temporal regions of the cortex were hypothesized to assume a similar function during the execution of isometric motor tasks performed at low intensity. Differences in the frontal and parietal lobes are thought to be associated with the brain’s electrical activity, which oscillates during the processing of attentional distractors when individuals execute attentionally demanding tasks (see Foxe & Snyder 2011 for a review).

## Method

### Participants

The institutional ethics committee of the first four authors approved the study. Undergraduate and postgraduate students were contacted via email and invited to participate. Those who expressed an interest were subjected to an initial survey to capture key demographic details (e.g. age and gender). The inclusion criteria were that potential participants were healthy and right-handed. Sample size was calculated by use of G*Power 3.1 and based on a large effect size of sensory modulation on attentional focus (*f* = 1; Hutchinson et al. 2015), 15 participants were required. Four additional participants were recruited to protect the study against participant attrition and deletions due to outliers. Accordingly, the sample comprised 19 participants (9 women and 10 men; *M*_age_ = 26.4, SD = 3.6 years; *M*_height_ = 170.4, SD = 9.5 cm; *M*_weight_ = 67.1, SD = 11.6 kg).

### Experimental procedures

A researcher initially explained the psychometric measures and addressed any queries from participants. He also cleaned the participant’s legs and face with preparation pads saturated with 70% isopropyl alcohol. Thereafter, five EMG surface electrodes (Goldy Karaya Gel electrodes, 28 mm diameter, silver/silver chloride, Arbo, Henley Medical, Stevenage, UK) were placed on the participant’s right leg, and 64 electroencephalography (EEG) electrodes (Quik Gel; Compumedics Neuromedical Supplies) were placed on the participant’s scalp. Each participant was asked to perform isometric ankle-dorsiflexion contractions at 20% of their maximal voluntary contraction (MVC).

A force transducer (Model 615, S-Type Load Cell, Tedea-Huntleigh Electronics, UK, max 100 kg) was used to measure the foot pressure produced. The participant was able to observe the strength line (Spike 2 v4.11; Cambridge Electronic Design) as a means to apply the required force. The maximum voluntary contraction (MVC) was assessed three times in order to identify the peak value prior to commencement of the experimental exercise bouts. The participant was asked to perform a maximal ankle-dorsiflexion contraction for 5 s, and there was a 2-min interval in between each attempt in order to negate the effects of fatigue on task performance. The force signal was amplified 1000 times, low-pass filtered at 2 KHz, and digitized at 1 KHz.

The experiment consisted of five sets of 30 trials (total = 150 trials). Each trial consisted of 10 s of contraction followed by 10 s of rest, and each participant was instructed to control the length and intensity of contraction by following the time and strength line, respectively. The sets could only recommence when the participant fully recovered, which was objectively assessed by means of the MVC and level of limb discomfort (CR10; Borg 1982). A short musical excerpt (2.8 s of the chorus of *Fancy* by Iggy Azalea, feat. Charli XCX. 95 bpm, 75 dBA) was used as an auditory distraction and to *possibly* degrade task performance levels. The musical excerpt was delivered by use of noise-cancelling headphones (Sennheiser HD201).

Two experimental conditions were administered: auditory distraction (AD; auditory stimulus applied during exercise) and stimulus-only (SO; auditory stimulus applied at rest), in addition to a control condition (CO; no intervention). During the first 20 trials of the set, AD and CO were randomized, as well as the moment at which AD started, which varied across 3, 4, and 5 s following initiation of the contraction. This approach was adopted in order to circumvent the potential confound of expectation. During the last 10 trials of the set, SO was used at random times as a means by which to isolate effects of the auditory stimulus that were not associated with exercise attentional shifts. E-Prime 2.0 was used to design the present experiment and deliver the auditory stimulus. The electronic devices were synchronized by use of a parallel port and the stimulus triggered an immediate mark in the EEG signal; the stimulus was subsequently used to epoch and average the trials (see “Data analysis” section).

#### Electromyography

Muscle electrical activity was measured by use of electromyography (EMG), which identifies the electrical potential generated by muscle cells. Surface electrodes were placed on the tibialis anterior and lateral gastrocnemius in accord with the recommendations of the SENIAM project (Surface Electromyography for the Non-Invasive Assessment of Muscles; Stegeman & Hermens 1999). The ground electrode was placed on the lateral malleolus. The EMG signal was amplified 1000 times, low-pass filtered at 20 Hz, and digitized at 1 KHz.

#### Electroencephalography

Brain electrical activity was assessed by means of a 64-channel Quik-cap. The 64 Ag/AgCl electrodes were attached to the participant’s scalp based on the international 10–20 system and filled with Quik gel (Compumedics Neuromedical Supplies). The mastoids were used to digitally reference the brain electrical signal. Two pairs of electrodes captured the horizontal and vertical eye movements. Impedance was kept below 5 kΩ. The brain electrical signal was amplified at a gain of 1000. Online bandpass filters 0.1–100 Hz were used to avoid electrical interference and muscle artefacts. The signal was acquired through the use of the software Scan 4.4 acquisition and digitized at 1000 Hz using Synamps amplifier (Compumedics Neuroscan).

### Data analysis

Spike2 (v4.11; Cambridge Electronic Design) was used to obtain time domain indices (root mean square; RMS) from the muscle electrical signal, which was initially filtered, rectified, and smoothed (Altimari et al. 2012). The RMS value obtained from the EMG data is representative of the motor units necessary to produce a given level of contractile force (Farina, Fosci, & Merletti 2002). The electrical activity in the anterior tibialis was used to quantify the influence of auditory distraction on motor unit recruitment during each trial.

Bad EEG electrodes were visually checked and those not working reliably were discarded before epoching methods. Independent component analysis was used to remove eye blinks by tracking down the activity of vertical eye movements (Zhou & Gotman 2009). The raw data were imported into the database by epoching the original file into 4.5-s windows (0.5 s before and 4 s after the auditory stimulus onset). The signal was DC-offset corrected in order to prevent the influence of voltage imbalance problems. Subsequently, the electrical signal in the brain was submitted to bandpass filters 0.5–30 Hz, 24 dB/octave and the time–amplitude signals were averaged for each experimental condition (i.e. grand average). The head model was computed through the use of OpenMEEG (Gramfort, Papadopoulo, Olivi, & Clerc 2010) based on the EEG cap that was used. The source of the brain electrical signal was reconstructed by applying the Minimum Norm Method (wMNE; output mode: Kernel; Pinto & Silva 2007). The source orientation was unconstrained, meaning that each vertex of the cortex surface contained three dipoles with orthogonal directions. This anatomical observation is based on the premise that neurons are not only organized in macro-columns perpendicular to the cortex surface. The signal-to-noise ratio was conventionally set at 3. The computed sources were averaged across participants, and the Mindboggle Atlas (Klein & Hirsch 2005) was used to identify the brain regions associated with the activity.

The source reconstruction was initially performed using full-frequency spectrum analysis. The results are presented for group data ensemble-averaged waveforms. A magnitude threshold was used to localize the sources of the brain electrical activity at approximately 35% of the peak (Jain, Gourab, Schindler-Ivens, & Schmit 2013). The EEG procedures were performed by use of Brainstorm (Tadel, Baillet, Mosher, Pantazis, & Leahy 2011), which is freely downloadable under the GNU public license (http://neuroimage.usc.edu/brainstorm). The EMG and EEG signals were compared using a paired samples *t* test (controlled over time dimensions) on Brainstorm, and the *p* value thresholds were corrected dynamically for multiple comparisons using the Bonferroni method. This method allowed the research team to compare the entire epoch across conditions and delineate the overall dimension of the waveform in the averaged samples (i.e. all peaks).

## Results

### Electrical activity in the muscle

**Fig. 1:**
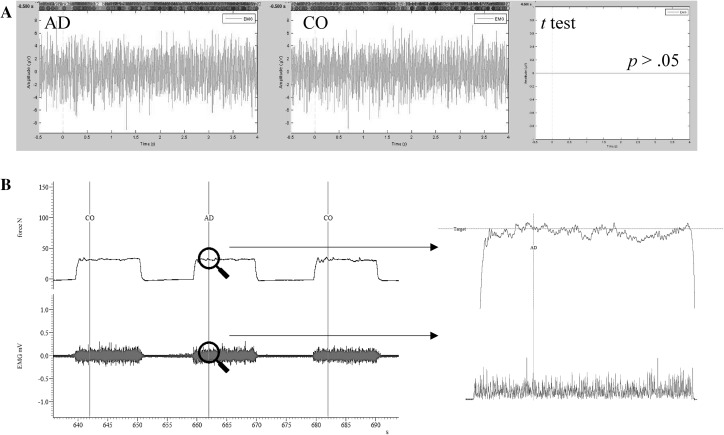
Comparison of EMG activity between AD and CO. *AD* auditory distraction, *CO* control; *Row A* grand average waveforms compared between AD and CO; *Row B* force and raw EMG data of participant 1 across three exercise trials

The electrical activity in the muscles produced by participants was used as an index of attentional distraction. Figure 1 serves to illustrate three experimental trials in the absence and presence of the auditory stimulus and the grand average for both conditions, where EMG data were compared by use of paired samples *t* tests. Results indicated that no statistical differences existed between AD and CO (*p* > .05), meaning that the auditory stimulus was not sufficient to have a detrimental effect on motor unit recruitment as hypothesized herein (see Fig. 1). A magnifying glass approach was used to zoom in on the force produced, and the electrical activity in the anterior tibialis at the moment the auditory stimulus was introduced. The electrical activity in the anterior tibialis was not affected by the attentional distraction. No statistical differences existed between AD and SO when the exercise trials were epoched and averaged.

### Electrical activity in the brain

**Fig. 2:**
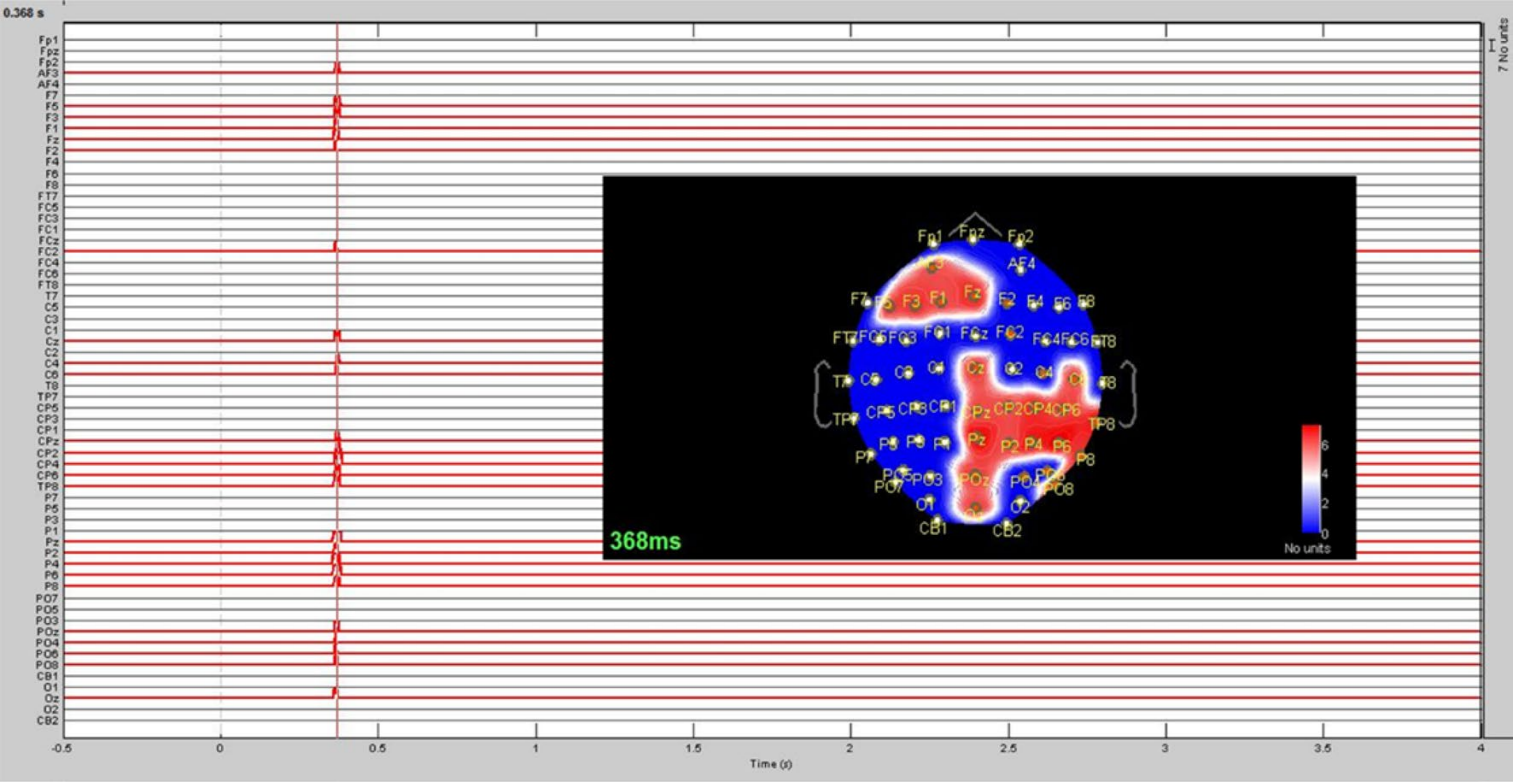
Paired samples *t* test comparing AD and SO. Spikes in the graph indicate statistically significant differences between conditions. A 2-D topographical map was created to anatomically localize the differences on the cortex surface. *AD* auditory distraction; *SO* Stimulus-only

**Fig. 3:**
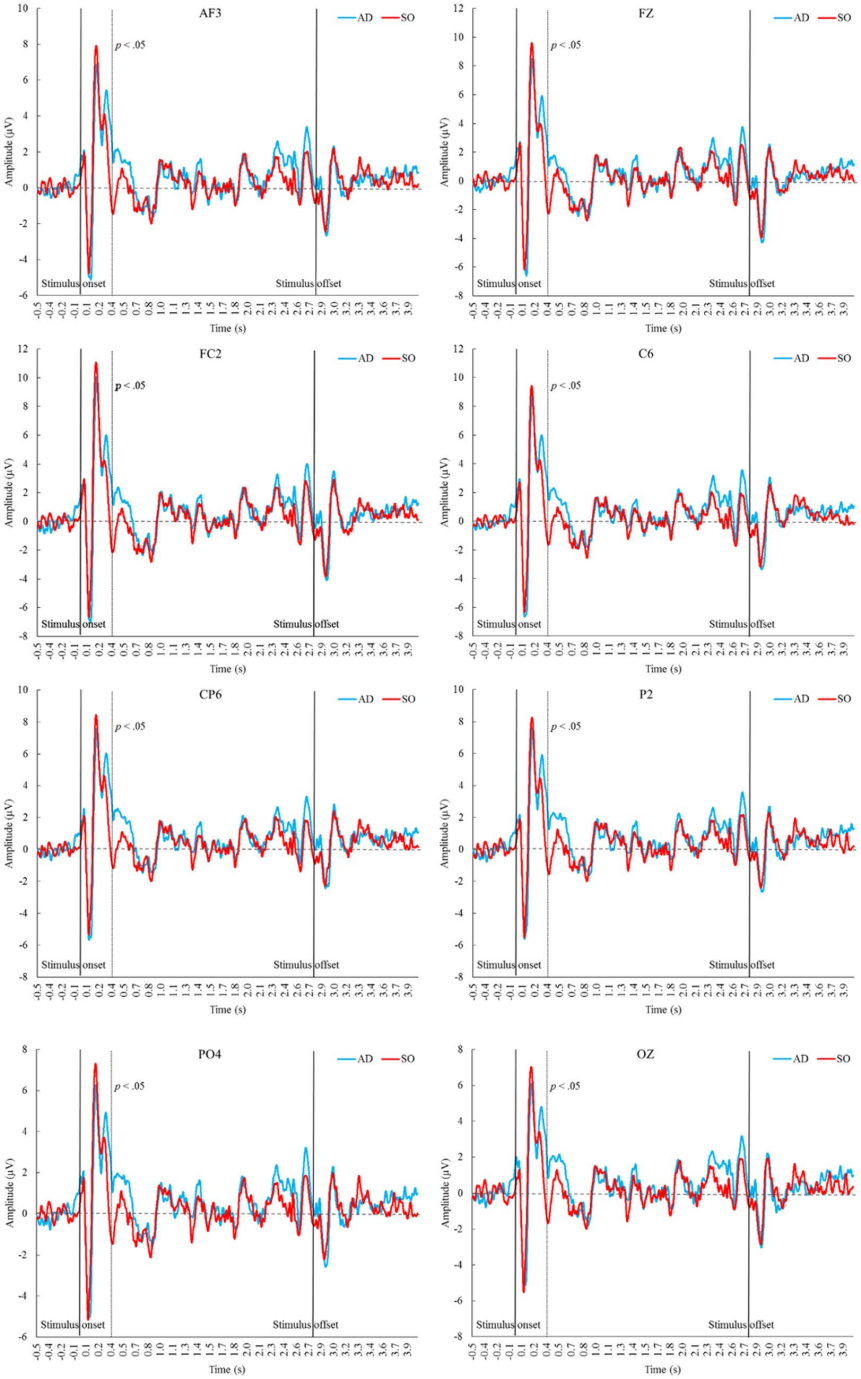
Grand average waveforms recorded at AF3, Fz, FC2, C6, CP6, P2, PO4, and Oz electrode sites presented for AD and SO. *AD* auditory distraction; *SO* stimulus-only

In comparing AD and SO, the research team expected to identify the electrode sites that activated in response to the processing of auditory distractions during the execution of a motor task. Accordingly, task-related information (i.e. pertaining to control and execution) was the only methodological factor responsible for inducing statistical differences in the EEG waveform pattern. Statistically significant differences were identified in the left frontal, central, central parietal, right parietal, and parietal-occipital regions of the cortex (Fig. 2; *p* < .05). A very similar EEG waveform pattern was identified in the right posterior/central (CPZ, CP2, CP4, CP6, TP8, PZ, P2, P4, P6, P8, POZ, PO4, PO6, PO8, and OZ) and anterior (AF3, F5, F3, F1, Fz, F2, and FC2) electrodes. Statistically significant differences between AD and SO occurred approximately 0.360 s after the stimulus onset and are possibly associated with the attentional demands imposed by sensory stimuli during the execution of motor tasks. The presence of task-related factors modulated N2 at 0.368 s after the stimulus onset. The signal amplitude in AD remained positive at approximately 0.360 s, while a sharp decrease was identified in the absence of muscular contractions (SO). The evoked potentials are presented in Fig. 3 for some of the electrode sites in which statistical differences were identified between AD and SO.

**Fig. 4:**
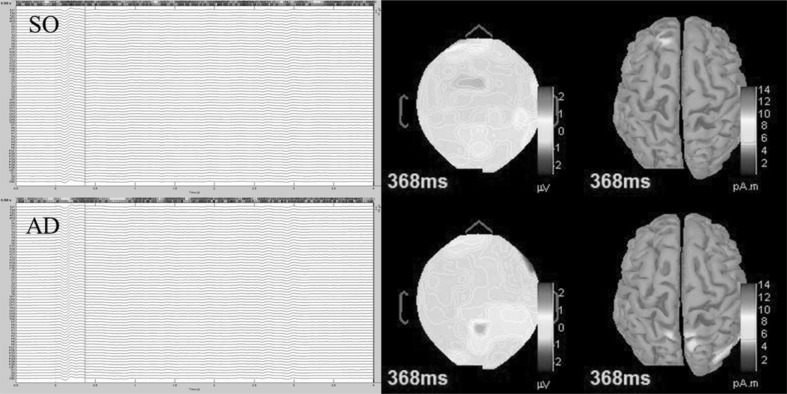
The reconstructed sources of the brain electrical activity for AD and SO at 0.368 s. Mindboggle Atlas was used to identify active brain regions. *AD* auditory distraction; *SO* stimulus-only

The distributed current source maps were reconstructed at 0.368 s (*p* < .05). The 2-D topography maps and estimated sources represent the group averaged data. The sources of the brain electrical signal indicated a conspicuous difference between anterior and posterior regions of the cortex. The presence of task-related factors increased the activity of the inferior and posterior parietal gyri at 0.368 s after the onset of the stimulus. The Mindboggle Atlas was used to locate and identify the brain regions that activated in response to attentional processing. Activity in the left superior frontal gyrus was evident when the stimulus was delivered in the absence of muscular contractions. Task-related factors reallocated the brain activity from left anterior to right and central posterior regions of the brain at 0.368 s after the stimulus onset (see Fig. 4).

## Discussion

This experiment attempted to further understand the neural systems that activate in response to auditory stimuli during the execution of an isometric ankle-dorsiflexion task. A brief musical excerpt was used to draw participants’ attentional focus toward task-irrelevant stimuli. In line with Rejeski’s (1985) conceptualization, the research team expected a parallel processing mechanism to emerge given that participants were required to monitor a range of task-relevant factors, such as the force generated and work duration (10 s of contraction and rest). Accordingly, the alerting system (i.e. immediate reallocation of attentional focus to the auditory stimulus; Fan, McCandliss, Fossella, Flombaum, & Posner 2005) would influence selective attention. Nonetheless, parallel information processing was expected to have immediate bearing over attentional focus in order to prevent likely detriments in neural activation of the working muscles.

The present results appear to uphold the veracity of Rejeski’s (1985) and Tenenbaum’s (2001) theoretical propositions, as parallel channels partially suppressed the processing of task-irrelevant signals, allowing participants to focus more intently on the task at hand. Moreover, the findings indicate that exercise complexity and intensity could have similar effects on selective attention, given that a distractive auditory stimulus was not sufficiently potent to disrupt task performance. In such instances, the frontoparietal network appears to activate at ~360 ms following stimulus onset. This is a means by which task-unrelated signals can be blocked and the neural activation of working muscles can be maintained. Nonetheless, auditory stimuli could have a more potent effect on the execution of low-demanding cognitive-motor tasks such as self-paced walking. Everyday tasks performed at a light intensity only require partial awareness to be executed successfully, meaning that environmental sensory stimuli have a strong bearing on attentional focus, which subsequently forces individuals to rearrange the motor plan (e.g. Haga et al. 2015).

According to Broadbent’s (1958) theoretical proposition, low-demanding cognitive tasks leave greater capacity for parallel processing, and thus, there is a reduced likelihood of task disruption to a primary task. Interestingly, recent evidence indicates that even walking tasks can be negatively affected by the presence of environmental distractions (e.g. smartphones), leading to detriments in task performance (Haga et al. 2015; Vredeveldt & Perfect 2014). This is predicated on the notion that low-demanding cognitive tasks only require partial awareness to be executed, leaving scope for environmental distractions to guide attentional focus toward task-irrelevant cues. Attentional shifts that are prompted by the presence of internal and external sensory cues appear to force the prefrontal cortex to inhibit inappropriate actions and maintain the motor plan. Nonetheless, this proposed mechanism does not appear to prevent auditory stimuli from entering focal awareness and disrupting task performance (e.g. during walking; Takeuchi et al. 2016).

### Electrical activity in the muscle

The electrical activity produced by the anterior tibialis was assessed in order to identify the likely negative effects of the auditory stimulus on neural activation and voluntary control. Electrical activity in the muscle was used as the primary index of attentional distraction given that the experimental task only required participants to contract the anterior tibialis at 20% of MVC. Minimal differences caused by attentional distractions should have elicited immediate changes in the neural control of movements with subsequent influence on EMG activity. Accordingly, a hypothetical decrease in the recruitment of motor units (i.e. measured by RMS) caused by the auditory stimulus would indicate that participants were only partially capable of processing task-irrelevant information during the execution of an isometric ankle-dorsiflexion task performed at low intensity (Petersen & Posner 2012). However, EMG signals were not influenced by the auditory stimuli, indicating that the immediate electrical signals evoked by the stimuli were rapidly inhibited via the mechanism of attentional suppression (Geng 2014) or parallel processed by alternative brain networks (Caputo & Guerra 1998).

The number of task-related factors can also influence the attentional system (Lavie et al. 2004). For example, if the motor task involves fine motor control of movements and high levels of concentration, even insignificant sensory stimuli can compromise the neural activation of the working muscles (see Bernstein & Bernstein 2015). Fortunately, the attentional system is trainable, and humans have developed psychological techniques that normally involve the control of physiological indices as a means by which to avoid the detrimental effects of task-irrelevant factors on task performance (Bernier et al. 2011; Desbordes et al. 2012). Tenenbaum (2001) suggested that exercise intensity can moderate the processing of environmental sensory stimuli. For example, whole-body exercises performed at high intensity force attentional focus toward interoceptive sensory cues and increase the prevalence of associative thoughts. In such instances, task-irrelevant factors remain outside of focal awareness because the brain has limited the capacity to process signals from multiple sources. It is noteworthy that the execution of repetitive movements appears to reallocate the organism’s attentional resources in accord with the relevance of both internal and external sensory stimuli; a potential confound that Broadbent (1958) did not contemplate in his original theoretical contribution.

### Cerebral responses

Electrical activity in the brain was compared primarily between AD and SO; thus, only task-related factors could be responsible for the differences in the evoked potential. Luck, Woodman, & Vogel (2000) pointed out that attentional processes would only suppress perceptual pathways if the sensory system is overloaded. In the present study, a highly demanding cognitive-motor task was used as a means by which to guide attentional focus toward task-relevant information. Statistically significant differences were identified in the left frontal, frontal-central, central, central parietal, right parietal, parietal-occipital, and occipital regions of the cortex. The presence of task-related factors (e.g. executing the motor task and monitoring the level of force produced) modulated N2 at 0.368 s after the onset of the stimulus. Such differences can be attributed to a parallel processing mechanism that initially occurred in the superior and inferior parietal regions of the cortex (Corbetta & Shulman 2002; Katsuki & Constantinidis 2014; Lee et al. 2013). No statistical differences were identified *during* the stimulus cessation; we contend that this cerebral response occurred owing to the fact that the auditory stimulus had already been partially suppressed approximately 0.360 s after the stimulus onset (Berti & Schröger 2003; Polich 2007). Therefore, the stimulus cessation would not have differed between AD and SO.

The high or low activity in the parietal lobe is primarily influenced by the number of task-related factors (Yin et al. 2012). The control of produced force and time duration serve to reallocate one’s attentional focus to task-related information. Irrelevant auditory stimuli are therefore supposed to force one’s attentional focus toward sensory pathways. This reallocation of attentional focus could possibly explain the differences in N2. Time domain analysis indicated that the presence of task-related factors prevented the sharp decrease of the EEG activity after approximately 0.360 s.

Auditory distractions have been commonly associated with changes in P300. Previous authors have suggested that up/down modulations in the time-series waveform that occur at ~350 ms following stimulus onset are induced primarily by stimulus-driven attentional processes originated in the frontal cortex (Berti & Schröger 2003; Polich 2007). There is evidence emerging to suggest that up modulations in P300 reflect a direct response to stimulus evaluation and decision-making processes (see Nieuwenhuis, Aston-Jones, & Cohen 2005; Twomey et al. 2015). P300 amplitude tends to increase during NoGo tasks (i.e. requiring self-control to elicit successful outcomes) as a form of response inhibition (Salisbury, Griggs, Shenton, & McCarley 2004), and similar responses have been successfully replicated in social contexts (see Nash, Schiller, Gianotti, Baumgartner, & Knoch 2013). Accordingly, the neural faculties and cognitive processes associated with up/down modulations in P300 appear to be far more complex than previously thought.

The results of the present study are in line with the extant literature (Linden 2005; Wang, Zheng, Zheng, & Sun 2015). We believe that changes in AD could have been caused by the presence of task-related factors, such as afferent feedback from working muscles and performance-related information, such as visual feedback (Vredeveldt & Perfect 2014). Up-regulation of the EEG waveform at ~ 360 ms following stimulus onset might be indicative of a swift decision strategy to reduce processing of potential distractors (for review, see Linden 2005). We hypothesize that up modulations at ~360 ms following stimulus onset could represent neurophysiological mechanisms that underlie Rejeski’s (1985) and Tenenbaum’s (2001) theoretical propositions. This electrophysiological response would partially block task-irrelevant information from entering focal awareness and thus causing disruption to exercise performance.

We hypothesize that the parietal lobe initially evaluated and successively reduced the processing of irrelevant stimuli such as a distracting musical excerpt (cf. Suzuki & Gottlieb 2013). The frontal lobe possibly received the signals from the parietal regions of the cortex and initiated appropriate action (i.e. stimulus interpretation; see Chadick & Gazzaley 2011; Prado, Carp, & Weissman 2011). Reduced activity in the left frontal regions induced by the presence of task-related factors (see Fig. 3) is believed to be caused by the previous suppression of irrelevant information in the parietal lobe. In summary, motor tasks performed in the presence of sensory stimuli appear to activate the parietal-frontal pathways. The parietal cortex not only functions as an informant in the parietal-frontal neural connection, but also performs initial evaluation of sensory signals (Bisley & Goldberg 2010) and thus may partially suppress or enable future processing in the frontal lobe, not only at rest, but also during exercise-related situations.

## Limitations of the present study

We hypothesized that statistical differences at 0.368 s after stimulus onset were possibly associated with parallel processing-related mechanisms (cf. Rejeski, 1985) that activate as a means by which to reduce the influence of attentional distractions on motor control. The present experiment was designed to mitigate the influence of potential confounds such as arousal-related responses caused by the execution of movements (Svebak and Murgatroyd 1985). Three methods were applied to isolate attention-related mechanisms from arousal-induced changes: (a) use of light intensity exercise bouts performed at 20% of MVC for short periods of time and separated by 10-s rest periods, (b) delivery of an auditory stimulus at random times after 3, 4, or 5 s of the contraction onset, and (c) delivery of an auditory stimulus at the end of block sessions (i.e. after 20 contractions). Therefore, arousal-induced modulations should bear minimal influence upon ERPs.

The research team decided to use *Fancy* by Iggy Azalea as a means by which to demonstrate that real exercise modes could be partially compromised by external sensory cues such as the chorus of a popular song. Interestingly, the stimulus was fully inhibited/parallel processed, and no physiological effects were identified. However, affective and perceptual responses were not analysed in the present study; we believe that the repetition of the auditory stimulus might have caused negative effective responses during the execution of isometric motor tasks. Accordingly, the brain’s electrical activity would have been influenced by such negative psychological responses (independent interference). The repetitive nature of event-related potential studies is a common problem in neuroscience (Picton et al. 2000), but the neuroscience research team attempted to select a reasonable number of trials that would not only facilitate the acquisition of meaningful data but also avoid the negative influence of extreme repetition. It should also be highlighted that the experiment did not include a manipulation check by which to gauge participants’ notion of perceived relevance of the task-demand characteristics and auditory stimuli that were presented in the experimental conditions. Furthermore, the motor task used in the present study does not represent a real-world mode of exercise such as cycling or running. Whole-body modes of exercise usually involve an extensive control of movements (Novacheck 1998) and could potentially involve different brain networks, generating dissimilar ERPs.

Changes in exercise intensity and complexity could also induce different ERPs. However, it is important to point out that this is the first study to address the brain mechanisms that underlie parallel attentional processing during exercise and therefore the control of external interferences such as a complex motor tasks and powerful muscular contractions should be maximized. Future research might aim to replicate the design of the present study in order to investigate the brain networks that activate during the execution of exercise performed at moderate and high intensities (i.e. fatiguing tasks). Such work would extend the neuroscientific examination of Rejeski’s (1985) and Tenenbaum’s (2001) theoretical propositions.

## Conclusions

This experiment adopted a theory-based approach to address the attentional neural systems that activate in response to potential distractors during the execution of an isometric task. The present findings appear to support Rejeski’s (1985) and Tenenbaum’s (2001) models given that parallel channels partially inhibited the processing of environmental sensory cues, thus allowing participants to execute the motor task. The recruitment of motor units in the anterior tibialis was not affected by external sensory cues, meaning that processing of auditory distractions was possibly suppressed during the execution of a demanding motor task. This neural faculty might have been developed through the ages as a means by which to prevent the influence of task-irrelevant factors on motor performance and enable humans to maintain the control of a task. Motor tasks performed in the presence of irrelevant sensory stimuli appear to activate the parietal-frontal network (Fan et al. 2005) as indicated by source reconstruction analysis. The presence of task-related factors (e.g. need to execute movements at precise intensities) during a highly demanding cognitive-motor task moderated the sharp decrease of EEG activity through the entire brain surface after approximately 0.360 s of the onset of the stimulus. This neurophysiological response could be associated with a decision-making strategy to reduce processing of external influences and thus prevent task performance from being disrupted.
